# HIV patients dying on anti-tuberculosis treatment: are undiagnosed infections still a problem in French Guiana?

**DOI:** 10.1186/s13104-020-05054-w

**Published:** 2020-04-10

**Authors:** Mathieu Nacher, Antoine Adenis, Philippe Abboud, Felix Djossou, Magalie Demar, Loïc Epelboin, Pierre Couppié

**Affiliations:** 1 Centre d’Investigation Clinique, CIC INSERM 1424, Centre Hospitalier de Cayenne, 97300 Cayenne, French Guiana; 2 Département de Formation et de Recherche Santé (DFR Santé), Université de Guyane, 97300 Cayenne, French Guiana; 3 Department of Infectious and Tropical Diseases, Centre Hospitalier de Cayenne, 97300 Cayenne, French Guiana; 4 UMR Tropical Biome and Immuno-pathophysiology, Université de Guyane, 97300 Cayenne, French Guiana; 5 Parasitology and Mycology Laboratory, Centre Hospitalier de Cayenne, 97300 Cayenne, French Guiana; 6 Department of Dermatology, Centre Hospitalier de Cayenne, 97300 Cayenne, French Guiana

**Keywords:** AIDS, Tuberculosis, Histoplasmosis, Death, Diagnosis, Latin America

## Abstract

**Objective:**

Despite scaling-up testing and antiretroviral treatment in Latin America, advanced HIV remains a significant public health problem. The objective of the present study was look for historical risk factors for death in French Guiana’s HIV cohort taking into account the immunological status, the main opportunistic infections, and their treatment. A retrospective cohort study was conducted on data collected between 1992 and 2008 to identify factors associated with death in a cohort 2323 patients.

**Results:**

There were 370 deaths for a total 9608 patient-years. Being on tuberculosis treatment was associated with a greater hazard of death. The diagnosis of confirmed tuberculosis, of histoplasmosis, of toxoplasmosis, and pneumocystosis were independently associated with death. Interactions terms between cotrimoxazole treatment and pneumocystosis, or between confirmed tuberculosis and tuberculosis treatment showed a protective treatment-effect. All patients having received anti-tuberculosis treatment (n = 347) did not have a final diagnosis of tuberculosis (n = 93). For histoplasmosis, 199 patients received antifungal treatment while 141 were diagnosed as having histoplasmosis. The number of patients on anti-tuberculosis drugs was far greater that the number of patients with confirmed tuberculosis, and these patients on treatment without confirmed tuberculosis had a twofold greater risk of dying.

## Introduction

Disseminated histoplasmosis and tuberculosis are 2 very common opportunistic infections of patients with advanced HIV disease in Latin America. It is not easy to differentiate them because rapid diagnostic tests for histoplasmosis are lacking in most of Latin America [1]. Histoplasmosis is hard to diagnose because it requires mycological expertise and because fungal growth is slow. Moreover, histoplasmosis is frequently associated with tuberculosis (ranging from 2 to 38% and in most countries > 10%) [2]. This diagnostic challenge is estimated to cause large numbers of deaths often attributed to tuberculosis [3–5]. It has been shown in Latin America that culture negative “tuberculosis” was associated with a 79% hazard increase [6]. The interpretation of this finding was that perhaps patients with culture-negative “tuberculosis” in fact had something else than tuberculosis. In French Guiana, the diagnosis of histoplasmosis relies on direct examination or culture, and clinicians are very aware of its presence, look for it aggressively and treat it presumptively whenever they suspect it, but they do not have routine access to antigen detection. With an incidence of 9.5 per 1000 person-years, tuberculosis is the 4th most frequent opportunistic infection among patients with HIV in French Guiana [7, 8]. Awareness of the presence of histoplasmosis started in the 1980s when the first cases of disseminated histoplasmosis with cutaneous and/or mucous lesions were diagnosed by dermatologists [9]. In the 1990s, following the introduction of fungal culture, the number of diagnoses greatly increased, and the number of deaths declined [10]. In a previous study we showed that disseminated histoplasmosis was the most frequent AIDS-defining infection in French Guiana [8]. In the present study, our objective was to identify historical risk factors for death, taking into account the immunological status, the main opportunistic infections [8], and their respective treatment.

## Main text

### Methods

We studied French Guiana’s HIV cohort data between 1992 and 2008 to search for factors associated with death in a cohort 2323 patients. HIV-infected persons in French Guiana receive free care and treatment. Foreigners also receive free care and treatment and residence permits for treatment. All antiretrovirals, genotyping, viral load, all diagnostic tests are available in French Guiana. For histoplasmosis, diagnosis rests on direct examination, fungal culture and histopathology [11]; for tuberculosis, Pasteur institute performs examination and culture, and molecular diagnosis. Details about this cohort have been presented elsewhere [12]. Trained research technicians routinely collect demographic, clinical and biological data from patient records in the 3 hospitals of French Guiana. Opportunistic infections, treatment of opportunistic infections, deaths, antiretroviral treatments, CD4 and CD8 counts are collected. Survival analysis was performed with death as the failure variable. The selection of variables was based on the use of background knowledge method [13]. The pertinent variables selected were the level of immunosuppression, antiretroviral treatment, the time period (because different time periods correspond to differences in the diagnostic and therapeutic arsenal), and the main opportunistic infections in French Guiana [8]. Crude incidence rates were obtained for different variable modalities. In this retrospective cohort, we used Cox proportional hazards modeling. Adjustments were made for categorized initial CD4 counts (< 200 CD4, 200-499, and > 499 CD4 per mm3), CD8 counts (< 643 (first quartile), > = 643 CD8 per mm3), age, time period (1992-1995 (pre-Highly Active Antiretroviral Treatment (HAART) and pre-fungal culture), 1996-2002 (post-HAART and post-fungal culture and pre-ambisome), and 2003-2008 (post-ambisome)), ARV treatment, confirmed histoplasmosis, histoplasmosis treatment (deoxycholate/liposomal amphotericin B and/or itraconazole), confirmed tuberculosis, tuberculosis treatment. Interaction terms were generated for each opportunistic infection and its treatment. Data were analyzed with STATA© 15.

### Results

Overall 2323 patients (1234 women and 1089 men) contributing 40,443 records were followed for a total 9608 person-years. The median follow-up duration was 2.9 years. The main nationalities were: French 499 (23.7%), Haitian 903 (42.8%), Guyanese 218 (10.3%), Surinamese 246 (11.7%), and Brazilian 167 (7.9%). The median age at censoring was 39 years (25%-75% quartiles = 31.07-48.07 years). At censoring the median CD4 count was 311 per mm3 (25%-75% quartiles = 121-497 CD4/mm3) and the median CD8 count was 849 per mm3 (25%-75% quartiles = 543-1238/mm3). At the time of the last observation 1251 persons were on antiretroviral treatment (53.8%).

There were a total of 370 deaths (3.85 deaths/100 person-years). The median time to death was 2.3 years (25%-75% quartiles = 0.8-4.5 years). The incidence rate of death varied between time periods: 14.6 deaths/100 person-years before 1997, 4.4 deaths/100 person-years for 1997-2002, and 1.5 deaths/100 person-years for 2003-2008. The model results are shown in Table 1. Being on tuberculosis treatment was associated with a greater hazard of death, adjusted Hazard Ratio (aHR) = 2.44 (95%CI 1.65-3.60)). The diagnoses of confirmed tuberculosis (aHR = 5.37 (95%CI 2.2912.58)), of histoplasmosis (aHR = 1.97 (95%CI 1.19-3.26)), of toxoplasmosis (aHR = 3.52 (95%CI 1.97-6.28)), and pneumocystosis (aHR = 4.18 (95%CI 1.48-11.80)) were significantly and independently associated with death. Esophageal candidiasis, one of the most frequent opportunistic infections, was not independently associated with death and removed from the model. When looking at interactions between cotrimoxazole treatment and pneumocystosis, or between confirmed tuberculosis and tuberculosis treatment, there was a significant protective effect. It is of note that all patients who received anti-tuberculosis treatment (n = 347) did not have a final diagnosis of tuberculosis (n = 93). For histoplasmosis, 199 patients received antifungal treatment while 141 were diagnosed as having histoplasmosis. The Cox model showed that the main opportunistic infections (except esophageal candidiasis, removed from final model) were independently associated with increased mortality (Table 1).

**Table 1:** Factors associated with death in a cohort of HIV-infected patients in French Guiana 19922008: multivariate Cox modeling

	Time at risk (person-years)	Incidence rate	Adjusted hazard ratio	95% confidence interval	P
Age < 30 years	1797	2.28	1		
Age [30-40 years]	2998	4.16	1.69	1.10-2.59	0.01
Age [40-60 years]	4116	3.76	1. 47	1.14-2.64	0.009
Age > 60	696	7	6.16	3.70-10.27	0.000
1991-1996 time-period	773	14.6	5.29	3.56-7.87	0.000
1997-2002 time-period	4182	4.47	2.68	1.96-3.68	0.000
2003-2008 time-period	4652	1.5	1		
CD8 < first quartile (643/mm^3^)	2156	7.2	1.77	1.37-2.28	0.000
CD8 >=643 per mm3	6716	2.09			
CD4 < 50	645	23.25	36.02	15.41-84.20	0.000
CD4 [50-200]	1809	5.14	12.34	5.32-28.63	0.000
CD4 [200-500]	4387	1.18	4.33	1.86-10.11	0.001
CD4 > 500	2167	0.27	1		
Antiretroviral treatment	4938	1.47	.30	0.22-0.40	0.000
No antiretroviral treatment	4670	6.35			
Tuberculosis (any location)	62	36.69	5.37	2.29-12.58	0.000
No tuberculosis	9546	3.63			
Tuberculosis treatment	238	24.3	2.44	1.65-3.60	0.000
No tuberculosis treatment	9370	3.32			
Interaction confirmed tuberculosis and tuberculosis treatment			0.20	0.07-0.60	0.004
Histoplasmosis	105	35.89	1.97	1.19-3.26	0.008
No histoplasmosis	9502	3.49			
Histoplasmosis treatment	289	7.2	.79	0.37-1.57	0.50
No histoplasmosis treatment	9319	3.74			
Interaction confirmed histoplasmosis and antifungal treatment			1.09	0.37-3.15	0.86
Pneumocystosis	70	17.06	4.18	1.48-11.80	0.007
No pneumocystosis	9538	3.75			
Cotrimoxazole	2230	6.77	1.44	1.08-193	0.01
No cotrimoxazole	7378	2.96			
Interaction pneumocystosis cotrimoxazole			0.13	0.03-0.49	0.003
Toxoplasmosis	86	44.14	3.52	1.97-6.28	0.000
No toxoplasmosis	9522	3.48			

### Discussion

The main opportunistic infections were associated with an increased risk of death after adjusting for CD4 count, age, period, and antiretroviral treatment. The interaction terms between treatment of tuberculosis and confirmed tuberculosis, and between treatment of pneumocystosis and confirmed pneumocystosis were logically associated with a decreased risk of dying. Cotrimoxazole treatment was independently associated with greater mortality, possibly because it reflected prophylaxis in advanced-HIV, but also because it reflected presumptive treatment. Tuberculosis treatment by itself was independently associated with an increased risk of death. Toxicities and drug–drug interactions may eventually lead to fatal outcomes. Nonetheless, another more plausible interpretation is that, in cases where the diagnosis is difficult, anti-tuberculosis treatment is given but that patients actually die from whatever disease caused TB-like symptoms.

Histoplasmosis is well known in French Guiana [14], nevertheless we may still be only seeing the upper part of the “iceberg”. The present results were adjusted for 3 time periods of increasing awareness and diagnostic capacity, which suggests that whatever the time period, patients on anti-tuberculosis treatment were at greater risk of dying. Despite our promptness to think about it, despite our fungal culture and pathology lab, we still may not have fully realized how common histoplasmosis is. Had we used antigen detection, we would have found even more cases and avoided unnecessary tuberculosis treatments thereby preventing more deaths. This humbling realization suggests that scaling up the availability of histoplasma antigen detection tests and lipoarabinomannan (LF-LAM) antigen tests is important for the care of patients with advanced HIV disease, even in a resource-rich country. More generally, although WHO has screening guidelines for advanced HIV, French HIV recommendations no longer cover the diagnosis of opportunistic infections in advanced HIV [15, 16]. However, patients are still often tested late with opportunistic infections. The evolution of the diagnostic arsenal and diagnostic and screening strategies should remain in HIV recommendations in order to obtain accurate diagnoses as early as possible and avoid fatalities due to undiagnosed pathogens.

## Limitations

In 2009 the data collection system changed and we were not able to get the information after that. In the decade since then, as the cascade of care improved, mortality gradually declined in HIV-infected patients but the context of death remains similar [17, 18]. Although anti-tuberculosis treatment is specific, antifungal treatment is not specific to histoplasmosis, which makes it more difficult to ensure it was prescribed for histoplasmosis. Despite these limitations, this hospital cohort data brings an interesting light to the challenges faced by clinicians and shows gaps in the HIV recommendations. Finally, the data analysis could not test some of the hypotheses regarding the observed associations with death.

## Data Availability

The anonymized data may be made available but this would require permission from the Commission Nationale Informatique et Libertés (CNIL), 3 Place de Fontenoy, 75007 Paris, France.

## Abbreviations

aHR: Adjusted hazard ratio
AIDS: Acquired Immuno Deficiency Syndrome
CD4: Cluster of differentiation 4
CD8: Cluster of differentiation 8
HAART: Highly active antiretroviral treatment
HIV: Human Immunodeficiency Virus
LF-LAM: Lateral flow lipoarabinomannan
TB: Tuberculosis
WHO: World Health Organization
